# *Artemisia gmelinii* Extract Attenuates Particulate Matter-Induced Neutrophilic Inflammation in a Mouse Model of Lung Injury

**DOI:** 10.3390/antiox12081591

**Published:** 2023-08-09

**Authors:** Hyeon-Ji Song, Dong-Uk Shin, Ji-Eun Eom, Kyung Min Lim, Eun Yeong Lim, Young In Kim, Ha-Jung Kim, Ju Hye Song, MyeongKuk Shim, HyeonJeong Choe, Gun-Dong Kim, So-Young Lee, Hee Soon Shin

**Affiliations:** 1 Division of Food Functionality Research, Korea Food Research Institute (KFRI), Wanju 55365, Republic of Korea; songhyeonji@kfri.re.kr (H.-J.S.); 50010@kfri.re.kr (D.-U.S.); jeeom@kfri.re.kr (J.-E.E.); limkyungmin@kfri.re.kr (K.M.L.); l.eunyeong@kfri.re.kr (E.Y.L.); k.youngin@kfri.re.kr (Y.I.K.); k.hajung@kfri.re.kr (H.-J.K.); s.juhye@kfri.re.kr (J.H.S.); kgd@kfri.re.kr (G.-D.K.); 2 Department of Food Science and Technology, Jeonbuk National University, Jeonju 54896, Republic of Korea; 3 Department of Food Biotechnology, Korea University of Science and Technology (UST), Daejeon 34113, Republic of Korea; 4 BL Healthcare Corp., Yongin 16827, Republic of Korea; qisi1212@bl-healthcare.com (M.S.); choehjung@bl-healthcare.com (H.C.)

**Keywords:** lung inflammation, respiratory disease, neutrophils, NETosis, NF-κB, MAPK, NRF2, HO-1

## Abstract

Particulate matter (PM) induces and augments oxidative stress and inflammation, leading to respiratory diseases. Although *Artemisia gmelinii* Weber ex Stechm has antioxidant and anti-inflammatory effects, there are no reports on whether *Artemisia gmelinii* extract (AGE) regulates lung inflammation in a PM-induced model. Thus, we investigated the protective effects of AGE using a PM-induced mouse lung inflammation model. AGE significantly decreased the expression of inflammatory chemokines, neutrophil extracellular trap formation, and the total number of inflammatory cells in the bronchoalveolar lavage fluid (BALF). Furthermore, AGE attenuated lung inflammation through the suppression of the nuclear factor kappa-light-chain-enhancer of activated B cells (NF-κB)/mitogen-activated protein kinase (MAPK) signaling pathway, while promoting the nuclear factor erythroid-2-related factor 2 (NRF2)/heme oxygenase-1 (HO-1) signaling pathway in lung tissues. Concordant with these observations, AGE suppressed inflammatory cytokines, chemokines, reactive oxygen species, NETosis, myeloperoxidase, and neutrophil elastase by decreasing the mRNA expression of High mobility group box 1, Runt-related transcription factor 1, and Kruppel-like factor 6 in differentiated HL-60 cells. In summary, our data demonstrated that AGE suppresses PM-induced neutrophil infiltration, lung damage, and pulmonary inflammation by suppressing NF-κB/MAPK signaling pathways and enhancing the NRF2/HO-1 signaling pathway. These findings suggest that AGE administration is an effective approach for preventing and treating PM-induced respiratory inflammation.

## 1. Introduction

In the last few decades, particulate matter (PM) has become a significant concern to human health, and its detrimental effects have become a major focus for governments and health organizations worldwide [1]. PM consists of solid and liquid droplets suspended in the atmosphere and can travel deep into the human alveolar region and penetrate the alveolar wall to the blood vessels, adversely affecting the respiratory, cardiovascular, and gastrointestinal systems [2,3,4]. According to the updated guidelines of the World Health Organization (WHO), the average annual concentration of PM should not exceed 5 µg/m^3^, and the daily average exposure should not exceed 15 µg/m^3^. If the level reaches ≥35 µg/m^3^ during a 24 h period, acute inflammation could be caused, and chronic exposure to levels above 50 µg/m^3^ can lead to severe health effects and even mortality [5]. Many pulmonary diseases, including asthma, chronic obstructive pulmonary disease (COPD), and acute lung injury (ALI), have been linked to PM exposure, resulting in inflammation [6,7,8,9]. Oxidative stress serves as a crucial molecular mechanism behind PM-induced lung damage and plays a significant role in the pathogenesis of various PM-induced human diseases [10].

Innate immune cells in the respiratory tract, such as macrophages and neutrophils, are the first to respond to inhaled pathogens. Many lung diseases, such as asthma, COPD, and ALI, are associated with chronic neutrophilic inflammation [11]. Neutrophils are recruited to the site of inflammation following stimulation by chemotactic factors released from damaged pulmonary tissues [12]. They utilize several host defense mechanisms, including phagocytosis, degranulation, reactive oxygen species (ROS), reactive nitrogen species production, pro-inflammatory cytokine production, and neutrophils extracellular trap (NET) formation. Several studies have showed that NET formation exacerbates pulmonary diseases, and NETs allow neutrophils to kill extracellular pathogens while minimizing damage to host cells. NETs are produced by the extracellular release of neutrophil nuclear material coated with antimicrobial peptides and enzymes and are primarily activated by ROS production [13]. NET formation further promotes the recruitment and activation of circulating neutrophils [14]. Therefore, the inhibition of PM-induced neutrophil activation may alleviate pulmonary diseases.

*Artemisia gmelinii* Weber ex Stechm (AG) is a plant species belonging to the Artemisia family, which includes over 500 plant species [15]. AG has been reported in Asia, including China, Mongolia, Russia, and Korea, to have medicinal properties for treating fever, coughs, headaches, colic, and digestive disorders [16]. It exerts various biological influences, such as antioxidant, anti-inflammatory, and anti-allergic effects [17,18,19]. In addition, our previous study showed that AG ethanol extract (AGE) decreased the release of inflammatory cytokines and chemokines, inhibited MAPK and NF-κB signaling pathways to alleviate lung inflammation in a COPD mouse model, and attenuated inflammation in cigarette smoke extract (CSE)/lipopolysaccharide (LPS)-treated mouse alveolar macrophages (MH-S) [20]. Nonetheless, the therapeutic effects of AGE on lung inflammation have yet to be demonstrated in a model of PM-induced lung injury. Hence, the objective of this study was to examine the impact of AGE using a mouse model of PM-induced lung injury, as well as a phorbol 12-myristate 13-acetate (PMA)-stimulated promyelocytic leukemia cell line (HL-60).

## 2. Materials and Methods

### 2.1. Materials

RPMI 1640, fetal bovine serum (FBS), penicillin–streptomycin, Hank’s balanced salt solution (HBSS), phosphate buffered saline (PBS), and Dulbecco’s phosphate buffered saline (DPBS) were purchased from Welgene (Gyeongsan-si, Republic of Korea), and PMA, hydrogen peroxide, dihydrorhodamine 123 (DHR123), 4′,6-diamidine-2′-phenylindole dihydrochloride (DAPI), and diemthyl sulfoxide (DMSO) were purchased from Sigma-Aldrich (St. Louis, MO, USA). WST-1 solution was obtained from Cellvia (Seoul, Republic of Korea). Q-plex assay kit was purchased from Quansys Biosciences (Logan, UT, USA), and Accustain T solution obtained from NanoEntek (Seoul, Republic of Korea). NEPER™ Nuclear and Cytoplasmic Extraction Reagent was purchased from Thermo Fisher Scientific (Waltham, MA, USA). We obtained p-p38, p38, p-p65, p65, p-IκB, IκB cyclooxygenase-2 (COX-2), nuclear factor erythroid-2-related factor 2 (NRF2), heme oxygenase-1 (HO-1), Lamin B1, β-actin, and anti-mouse or anti-rabbit IgG antibodies from Cell Signaling Technology (Danvers, MA, USA). ChemiDoc XRS+ was purchased from Bio-Rad (Hercules, CA, USA). SYTOX green was obtained from Invitrogen (Carlsbad, CA, USA). Myeloperoxidase (MPO) and neutrophil elastase (NE) enzyme-linked immunosorbent assay (ELISA) kits were obtained from Abcam (Cambridge, UK). MPO antibody was purchased from Santa Cruz Biotechnologies (Dallas, TX, USA). QIAzol Lysis Reagent and RNeasy Mini Kit were purchased from Qiagen (Valencia, CA, USA).

#### 2.1.1. Preparation of PM

The urban PM reference sample of the National Institute of Standard and Technology (NIST) (SRM 1648a) was purchased from Sigma-Aldrich (St. Louis, MO, USA). The particles were collected from St. Louis, Missouri, over 24 month and used as reference material. SRM 1648a contains a combination of polycyclic aromatic hydrocarbons (PAHs) and organic and inorganic pollutants. PM 10 mg/kg was dissolved in PBS for use in experiments.

#### 2.1.2. Plant Material and Preparation of Extracts

The AG used in this study was provided by BL healthcare Corp. (Cheongju-si, Republic of Korea). Dried AG was extracted with a 15× volume of 50% ethanol by incubating at ~50 °C for 6 h. AGE was subsequently filtered using Rotavapor R-210 (BUCHI Labortechnik AG, Flawil, Switzerland), concentrated under vacuum at 50 °C, dried, and stored at −80 °C until use. AGE was dissolved in PBS for the in vivo experiment and dissolved in DMSO for the in vitro experiment.

### 2.2. Animal Study

#### 2.2.1. PM-Induced Lung Injury Mouse Model and Experimental Design

Male BALB/c mice, approximately 5 weeks old and weighing around 20 g, were procured from Orient Bio (Gyeonggi, Korea). Following a 1-week acclimation period, the mice were randomly allocated into five groups: control (Naive, n = 5), PM-treated (PM, n = 5), PM and 3 mg/kg dexamethasone-treated (DEX, n = 5; serving as a positive control), PM and 100 mg/kg AGE-treated (100, n = 5), and PM and 200 mg/kg AGE-treated (200, n = 5) groups. To induce lung injury, the mice were given an intranasal administration of 20 μL of PM (10 mg/kg) five times a week from days 8 to 27. Mice in the PM, AGE 100, and 200 groups received an oral administration of 200 µL/mouse of either PBS or AGE, diluted in PBS, from days 0 to 27. Meanwhile, those in the DEX group were orally given 200 µL of dexamethasone, diluted in PBS, from days 8 to 27. On day 28, the mice were euthanized, and samples of BALF, blood, and lung tissue were collected for further analysis. All animal procedures were conducted in accordance with the Animal Use and Care Guidelines of the Korea Food Research Institute (approval number: KFRI-M-22060). The mice were housed under conventional conditions in a consistent environment, observing a regular 12 h light/dark cycle, with the temperature and relative humidity maintained at 23 ± 2 °C and 50 ± 5%, respectively. The mice were given unrestricted access to food and water.

#### 2.2.2. Analysis of Chemokines in BALF

Chemokines such as CXCL-1, MCP-1, MIP-1α, RANTES, and TARC in the BALF were evaluated using a Q-plex assay kit (Quansys Biosciences, Logan, UT, USA). In brief, a calibration curve was established by employing a series of 1:2 dilutions with known concentrations of various cytokines and chemokines. Fifty microliters of the calibrator and samples from each well were introduced into a Q-Plex^TM^ array 96-well plate and incubated for 1 h at room temperature. Following this, the plates were incubated with streptavidin–horseradish peroxidase and the substrate mixture at room temperature for 15 min. Ultimately, images were produced in the Q-View^TM^ Imager LS with an exposure time of 270 s. The concentrations of cytokines and chemokines were analyzed using the Q-View^TM^ software.

#### 2.2.3. Analysis of the Infiltration Cell Counts and Diff-Quick Stain in BALF

To quantify the total number of immune cells in the BALF, 10 μL of BALF was combined with 10 μL of Accustain T solution (NanoEntek, Seoul, Korea). Following this, 12 μL of the mixed solution was loaded into the Accuchip channel, and the ADAM-MC^TM^ automated cell counter was utilized to tally the total cell count. BALF (150 μL) was then applied to the coated slides, the immune cells were stained using the Diff-Quick staining reagent (38721l; SYSMEX, Kobe, Japan), and the numbers of macrophages and neutrophils were assessed under a microscope.

#### 2.2.4. Histological Analysis and Immunohistochemistry (IHC) Staining of the Lung Tissues

The left lung samples were immediately fixed in 10% formalin solution for 2 days at room temperature. After fixation, the lung specimens were dehydrated and embedded in paraffin. The lung tissues were then sectioned at 4.5 μm thickness. The lung tissues were stained with hematoxylin and eosin (H&E) and periodic acid–Schiff (PAS) to visualize the infiltration of inflammatory cells and the secretion of mucus around bronchus. To first measure small airway wall thickness, the distances were calculated from the luminal side to the outer edge of the airway wall at multiple points along the airway, with a preference for regular intervals. Additionally, the value of a small airway area was determined by subtracting the measured area of the inner edge from the total area of the small airway. The small airway thickness and area were then calculated by averaging the measurements of each group. Next, the severity of peri-bronchial inflammation was analyzed semi-quantitatively using a scale ranging from 0 to 5. The scores were assigned as follows: 0, no inflammation cells; 1, a few inflammatory cells; 2, a ring of inflammatory cells 1 cell-layer deep; 3, a ring of inflammatory cells 2 cells deep; 4, a ring of inflammatory cells 3–4 cells deep; and 5, a ring of inflammatory cells > 4 cells deep. Furthermore, we conducted a PAS-positive cell count. The lung tissues were stained with PAS stain to evaluate the mucus secretion in which goblet cells play the main role. The goblet cells were stained and appeared with purple color. The number of PAS-positive cells in the bronchus of lung tissues was counted using light microscopy at 200× magnification, which was based on counting nuclei with dark purple stained cytoplasm. We measured the area density of PAS-positive cells using FIJI application, a software tool used for image analysis.

For immunohistochemistry, the lung tissues were embedded in paraffin blocks and deparaffinized, and antigen retrieval was performed in a water bath for 20 min at 98 °C. The slides were then incubated in a 0.3% hydrogen peroxide solution for 5 min at room temperature to inhibit peroxidase activity. After washing, the slides were blocked with a goat serum-blocking solution and incubated with Ly6G primary antibodies (1:100) overnight at 4 °C. The next day, the slides were washed with TBST for 5 min and stained with the ABC reagent for 30 min. The slides were visualized using a 3,3′-diaminobenzidine (DAB) peroxide substrate kit (Vector Laboratories Inc., Burlingame, CA, USA) and counterstained with hematoxylin.

#### 2.2.5. Western Blot

Lung tissues were homogenized for protein extraction using a lung dissociation kit (Protein Simple, Santa Clara, CA, USA). The tissues were then lysed in an ice-cold lysis buffer containing protease inhibitors. The protein concentration in each sample was determined using the Bradford protein assay. An amount of 10 μg of the protein mixture from each sample was loaded into the wells of Mini-PROTEAN^®^TGX^TM^ Precast Gels. Following electrophoresis, the proteins were transferred onto Western blotting filter membranes for 60 min at 100 V. Post-transfer, the membranes were blocked with EveryBlot blocking buffer (Bio-Rad) for 20 min at room temperature. Subsequently, the membranes were incubated with diluted antibodies against p-p38, p38, p65, IκB, cyclooxygenase-2 (COX-2), nuclear factor erythroid-2-related factor 2 (NRF2), heme oxygenase-1 (HO-1), Lamin B1, and β-actin (Cell Signaling Technology, Danvers, MA, USA) overnight at 4 °C. The next day, the membranes were washed three times with TBST for 10 min each. After washing, the membranes were incubated with horseradish peroxidase-conjugated anti-mouse or anti-rabbit IgG antibodies for 1 h at room temperature and then washed three more times with TBST for 10 min each. Protein bands were quantified using a ChemiDoc XRS+ (Bio-Rad, Hercules, CA, USA).

### 2.3. HL-60 Cell Experiment

#### 2.3.1. Cell Culture and Differentiation

HL-60 human peripheral blood lymphoblast cells were obtained from the American Type Culture Collection (Manassas, VT, USA). The cells were cultured in RPMI 1640 medium containing 10% fetal bovine serum and 1% penicillin–streptomycin. They were differentiated in RPMI medium containing 1% dimethyl sulfoxide at a density of 2 × 10^5^ cells/mL for 5 days.

#### 2.3.2. Cell Viability

The WST-1 (Water-Soluble Tetrazolium 1) assay was performed to investigate cytotoxicity. Differentiated HL-60 (dHL-60) cells were seeded into 96-well plates and co-treated with 100 nM PMA and AGE (25, 50, and 100 µg/mL) at 37 °C for 3 h. The plates were then centrifuged at 311× *g* for 5 min to adhere the cells to the bottom of the plate, and the supernatants were collected to measure other factors. Next, WST-1 solution was mixed with the medium at a ratio of 1:10 and added to each well, and the plates were incubated at 37 °C for 30 min. The absorbance at 450 nm of each well was measured using an Epoch microplate reader (BioTek, Winooski, VT, USA).

#### 2.3.3. NETosis Assay

For the NETosis assay, dHL-60 cells were seeded into 96-well black plates at a density of 1 × 10^5^ cells/well and co-treated with 100 nM PMA (Sigma Aldrich, St. Louis, MO, USA) and 25, 50, and 100 µg/mL AGE at 37 °C for 3 h. The plates were centrifuged at 311× *g* for 5 min, and the supernatants were harvested to measure the level of MPO and NE using an ELISA Kit (Abcam, Cambridge, UK) following the manufacturer’s instructions. The cells were then treated with 5 µM SYTOX green, an extracellular DNA dye (Invitrogen, Carlsbad, CA, USA), which was diluted using Hank’s balanced salt solution (HBSS). The cells were incubated for 10 min in the dark. Fluorescence intensities were measured using SpectraMax i3 (Molecular Devices, San Jose, CA, USA) at 480/520 nm.

#### 2.3.4. ROS Production Assay

To assess ROS production, dHL-60 cells were seeded into 96-well V-bottom plates at a density of 1 × 10^5^ cells/well and co-treated with 100 nM PMA and 25, 50, and 100 µg/mL AGE at 37 °C for 20 min. The cells were then centrifuged at 311× *g* for 5 min, the supernatants were removed, and the cells were washed with HBSS. The washed cells were treated with 5 µM DHR123 (Sigma Aldrich, St. Louis, MO, USA), a non-fluorescent membrane-permeable indicator of ROS. DHR123 undergoes oxidation to its fluorescent form, rhodamine 123, indicating the presence of ROS. The cells were incubated with DHR123 for 20 min, and then washed with HBSS. Next, the cells were transferred to a 96-well black plate. The fluorescence intensity was measured at 500/530 nm using a SpectraMax i3 plate reader to quality the levels of ROS (Molecular Devices, San Jose, CA, USA).

#### 2.3.5. Visualization of NETs

To visually measure NETosis, dHL-60 cells were seeded onto Collagen I 8-well culture slides, co-treated with 100 nM PMA and 100 g/mL AGE for 3 h, and centrifuged at 311× *g* for 5 min, after which the supernatants were removed. The cells were then fixed with 4% formaldehyde for 15 min and permeabilized with 0.2% Triton X-100 for 5 min at room temperature. After washing with DPBS, the cells were blocked for non-specific binding with TBST containing 3% bovine serum albumin (BSA) for 1 h and then incubated with diluted anti-MPO (Santa Cruz Biotechnologies, Dallas, TX, USA) overnight at 4 °C. The following day, the cells were washed 3 times with TBST and stained with goat anti-mouse IgG antibody conjugated with Alexa Flour 488 (Invitrogen, Carlsbad, CA, USA) for 2 h at room temperature in the dark. The DAPI solution was then added to the cells. Confocal fluorescence imaging was performed using FV3000 Confocal Laser Scanning Microscope (Olympus, Tokyo, Japan).

#### 2.3.6. Visualization of Cytosolic ROS Production

For determining cytosolic ROS production, dHL-60 cells were seeded onto Collagen I 8-well culture slides and incubated for 30 min. After incubation, the cells were co-treated with 100 nM PMA and 100 µg/mL AGE for 20 min. The supernatant was removed, and the cells were fixed with 4% formaldehyde for 15 min and then permeabilized with 0.2% Triton X-100 for 5 min. After blocking the cells with 3% BSA, the cells were stained using 5 µM DHR123 overnight at 4 °C. The next day, the cells were washed thrice with TBST, and DAPI solution was added.

#### 2.3.7. Quantitative RT-PCR

The cells from each group were lysed using QIAzol Lysis Reagent, and RNA was extracted from the cells using an RNeasy Mini Kit (Qiagen, Valencia, CA, USA) according to the manufacturer’s protocol. The extracted RNA was quantified using an Epoch Microplate Spectrophotometer (BioTek, Winooski, VT, USA). cDNA was synthesized at 45 °C for 1 h, followed by 95 °C for 5 min using a C1000 Thermal Cycler. Next, qPCR was performed on a Step-One Plus Real-Time PCR System (Applied Biosystems, Foster City, CA, USA). Gene expression levels were normalized to those of housekeeping genes, and relative expression was calculated using the 2^−ΔΔCT^ method. The genes analyzed and the primer sequences are listed in Supplementary Table S1.

### 2.4. Statistical Analysis

Statistical analyses were performed using GraphPad Prism 9 software (version 9.0; La Jolla, CA, USA). The in vivo and in vitro data are expressed as mean ± standard deviation (SD) of independent experiments. Differences between groups were analyzed using one-way ANOVA, followed by Dunnett’s test, and the differences were considered significant at *p*-value < 0.05.

## 3. Results

### 3.1. Effect of AGE in the PM-Induced Lung Injury Mouse Model

**Figure 1:**
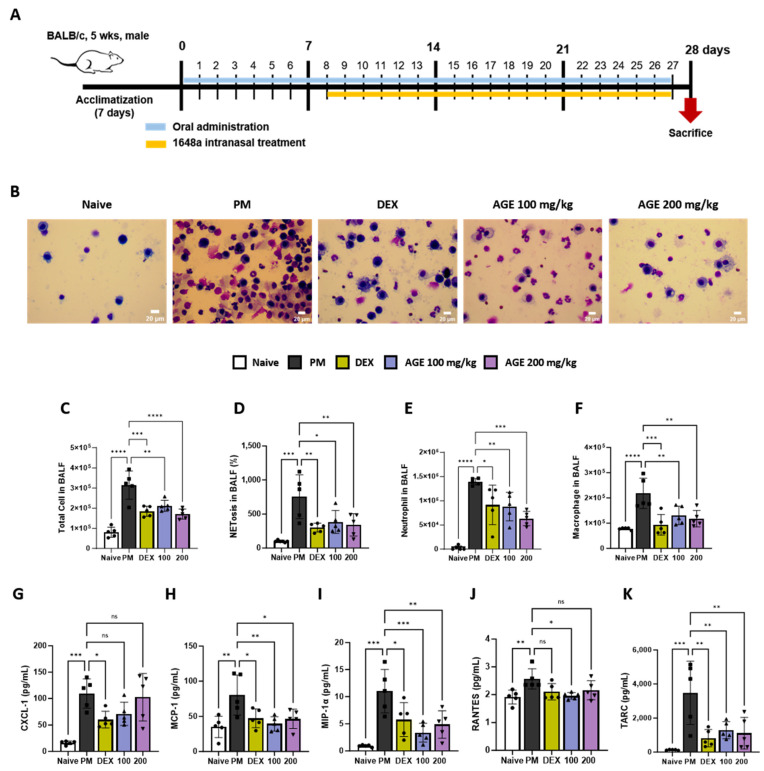
Effect of AGE on the inflammatory cell count and chemokines in BALF from the PM-induced lung injury mouse model. (**A**) Schematic of the PM-induced model timeline. (**B**) The inflammatory cell infiltration was measured using Diff-Quik staining methods. (**C**) Total cells in BALF counts using automatic cell counter. (**D**) NETosis in BALF stained with SYTOX green, and assessed using a fluorescence microplate reader. The numbers of (**E**) neutrophils and (**F**) macrophages in BALF were estimated using microscopy images. (**G**–**K**) The levels of chemokines (CXCL-1, MCP-1, MIP-1α, RANTES, and TARC) were measured using a Q-plex Assay Kit. Data were analyzed using one-way ANOVA and Dunnett’s test. All values have been reported as mean ± SD. ns: no significant, * *p* < 0.05, ** *p* < 0.01, *** *p* < 0.001, **** *p* < 0.0001. Naive (Circle): vehicle control, PM (Square): PM treatment, DEX (Hexagon): Dexamethasone (3 mg/kg) + PM treatment, 100 (Triangle): *Artemisia gmelinii* extract (100 mg/kg) + PM treatment, 200 (Inverted triangle): *Artemisia gmelinii* extract (200 mg/kg) + PM treatment.

We conducted an analysis of inflammatory cells, NETosis, and pro-inflammatory chemokines in the BALF (Figure 1A). The impacts of AGE on the infiltration of total cells, macrophages, and neutrophils in BALF were evaluated using Diff-Quik staining and microscopic examination (Figure 1B–E). In comparison to the naive group, the PM group displayed a significant increase in the number of total inflammatory cells, macrophages, and neutrophils in BALF. However, AGE treatment significantly decreased the number of infiltrating cells. Many studies showed that PM exposure stimulated neutrophils and increased NETosis [21,22]. In our study, PM treatment significantly increased NETosis in BALF. However, AGE treatment significantly decreased NETosis (Figure 1F). Additionally, the levels of pro-inflammatory chemokines, such as CXCL-1, MCP-1, MIP-1α, RANTES, and TARC, increased in the PM group. However, AGE treatment revealed markedly decreased levels of MCP-1, MIP-1α, and TARC, while CXCL-1 and RANTES levels showed a slight reduction compared to the PM group (Figure 1G–K).

### 3.2. Effect of AGE on the Protein Expression in PM-Stimulated Lung Tissues

**Figure 2:**
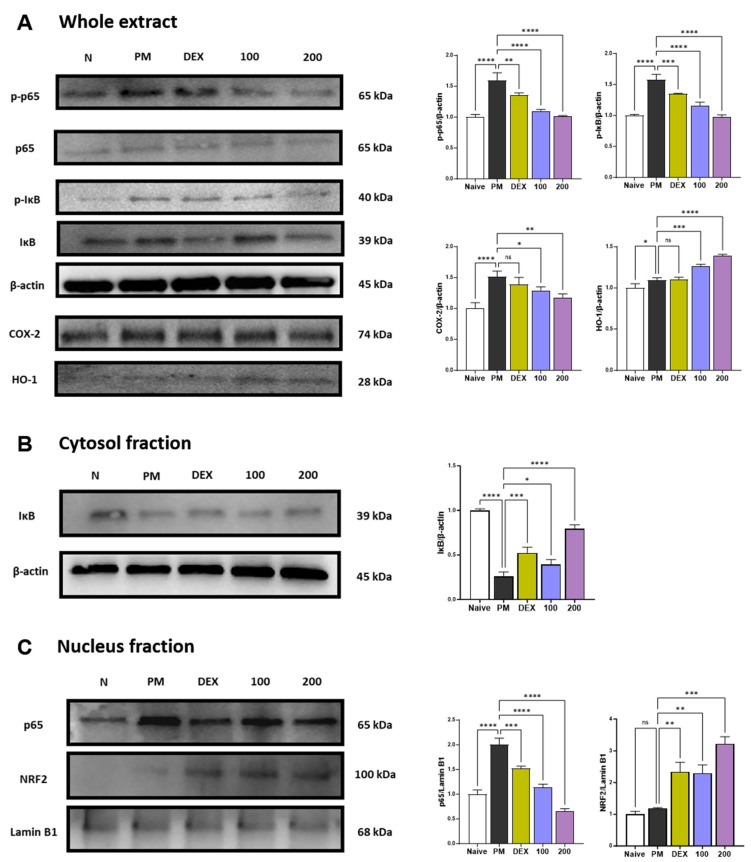
Effect of AGE on pro-inflammatory and antioxidant pathways in the lung tissues. (**A**–**C**) Protein expression levels of p-p38, p38, p-p65, p65, p-IκB, IκB, COX-2, NRF2, HO-1, Lamin B1, and β-actin. Data were analyzed by one-way ANOVA by Dunnett’s test. All values have been reported as mean ± SD. ns: no significant, * *p* < 0.05, ** *p* < 0.01, *** *p* < 0.001, **** *p* < 0.0001.

p38 MAPK and NF-κB p65 represent two transcription factors critically involved in inflammatory responses [23,24]. p38 MAPK and NF-κB can regulate the expression of chemokines related to neutrophils recruitment such as CXCL-1, MCP-1, MIP-1α, RANTES, and TARC [24,25]. p38 is extremely sensitive to redox reactions and is triggered by cytoplasmic ROS [26]. These pathways also regulate the expression of COX-2 [27]. PM treatment increased the phosphorylation of p38 (Figure S1) and the phosphorylation and translocation of NF-κB p65 (Figure 2A–C). Treatment with AGE 100 mg/kg has no effect on p38 phosphorylation, while AGE at 200 mg/kg significantly inhibited p38 phosphorylation (Figure S1). In the NF-κB pathway, AGE treatment significantly inhibited the phosphorylation and translocation of NF-κB p65 in a concentration-dependent manner. Thus, AGE, like dexamethasone, a positive control, alleviates PM-induced lung injury by suppressing the phosphorylation of p38 and the phosphorylation and translocation of NF-κB p65. PM stimulation is known to increase the expression of COX-2 [28,29,30]. COX-2 may also be a source of ROS [31], and these ROS are essential for the development of NETosis. Therefore, we determined whether AGE influenced the PM-induced expression of COX-2 protein. In the present study, PM significantly increased the expression of COX-2. However, AGE treatment decreased COX-2 protein expression in lung tissue in a concentration-dependent manner. Long-term exposure to PM causes oxidative stress in the lungs [32]. Previous studies demonstrated that HO-1 has antioxidant effects and attenuates lung damage [33,34]. Under oxidative stress, NRF2 translocates to the nucleus and binds to antioxidant response elements (ARE). After binding, NRF2 regulates HO-1 transcription. As shown in Figure 2C, AGE treatment significantly increased the expression of NRF2. Accordingly, the HO-1 expression increased in a concentration-dependent manner. Thus, we found that AGE exert antioxidant effects by regulating the NRF2/HO-1 pathway.

### 3.3. Effect of AGE on Histopathologic Analysis of the PM-Stimulated Lung Tissues

**Figure 3:**
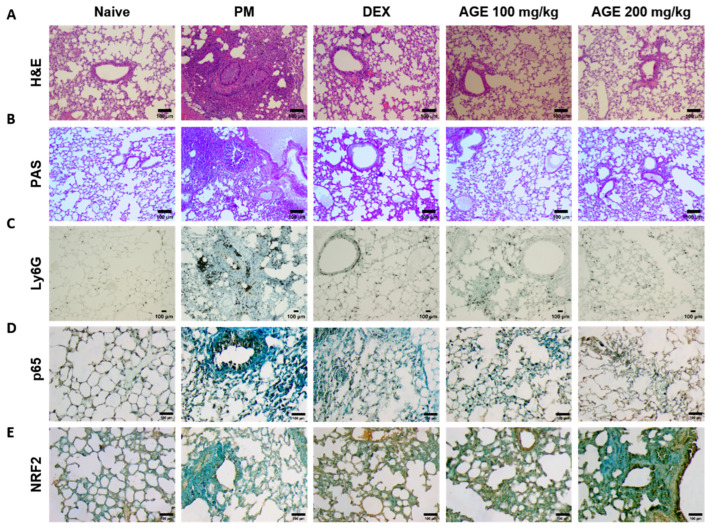

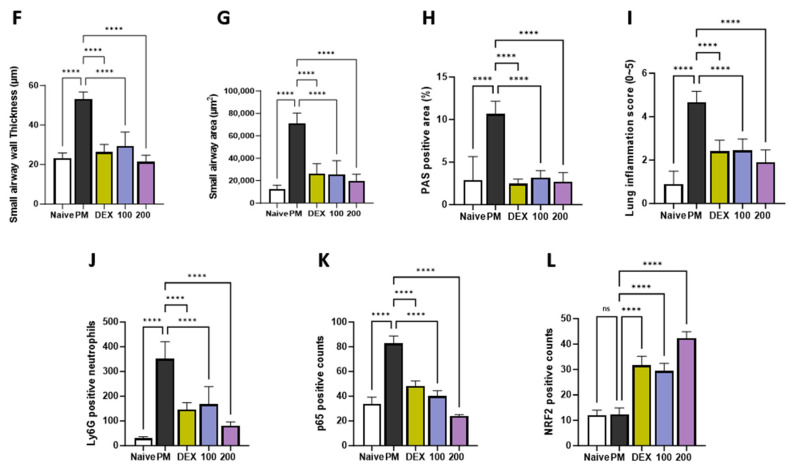
Effect of AGE on histopathological changes and neutrophils recruitment in the lung tissues. Lung tissues from each mouse group were collected and stained for (**A**) hematoxylin and eosin (H&E) staining, (**B**) periodic acid–Schiff (PAS) staining, and (**C**) Ly6G, (**D**) p65, and (**E**) NRF2 antibodies stained using immunohistochemistry (IHC) staining. Lung tissues from each mouse group were analyzed. (**F**) Small airway wall thickness (µm), (**G**) small airway area (µm^2^), (**H**) PAS-positive area (%), (**I**) lung inflammation score (0~5), (**J**) Ly6G-positive neutrophils, (**K**) p65-positive counts, and (**L**) NRF2-positive counts using FIJI application. Data were analyzed by one-way ANOVA by Dunnett’s test. All values have been reported as mean ± SD. ns: no significant, **** *p* < 0.0001.

To evaluate the histological effects of AGE on inflammatory cell infiltration and neutrophil recruitment in PM-treated mice, we performed hematoxylin and eosin (H&E), periodic acid–Schiff (PAS), and immunohistochemistry (IHC) staining in the lung tissues. H&E staining showed that the lung tissues of the PM-induced group were significantly infiltrated by inflammatory cells, leading to an increased enlargement of the alveolar space, airway inflammation, and thickness compared to those observed in the naïve group. In contrast, these infiltrations decreased in AGE-treated lung tissues (Figure 3A,F,G,I). The PM-treated group showed a significant PAS-positive area with goblet cell hyperplasia; however, treatment with AGE led to a decrease in mucus production and the proliferation of airway goblet cells (Figure 3B,H). Additionally, the PM-induced group showed a higher number of Ly6G-positive neutrophils compared to the naive group, whereas treatment with AGE decreased in neutrophil recruitment (Figure 3C,J). In the PM-induced group, the counts of p65 positive cells were higher compared to the naive group, while the AGE-treated group exhibited a decrease in p65-positive cell counts in the lung tissues (Figure 3D,K). Furthermore, the AGE-treated group significantly increased the counts of NRF2-positive cells compared to the PM-induced group (Figure 3E,L). These results demonstrate that AGE protects against PM-induced lung by decreasing the airway inflammation, mucus production, neutrophil recruitment, and oxidative stress.

### 3.4. Effect of AGE on NETs Released from dHL-60 Cells

**Figure 4:**
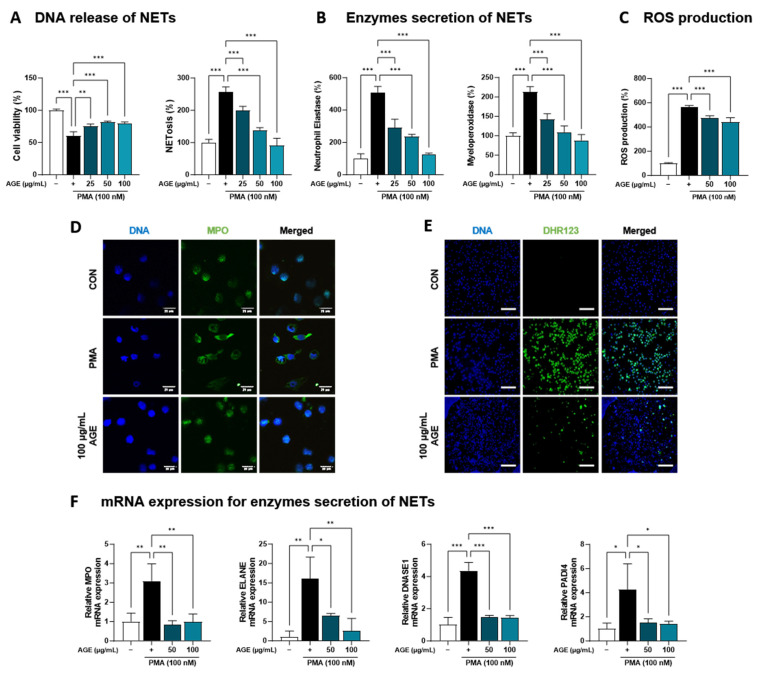

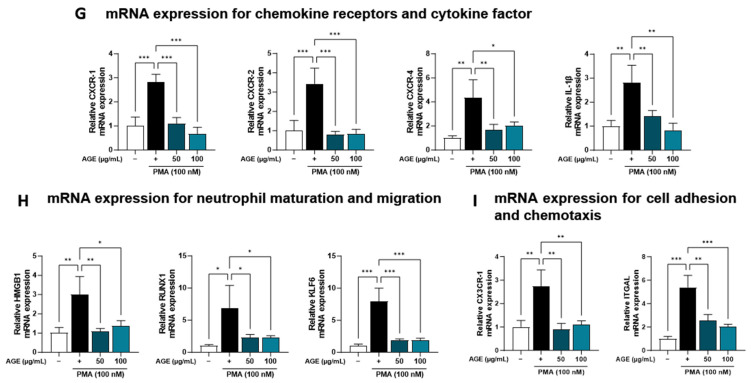
Effect of AGE on NETs formation and ROS production. (**A**) dHL-60 cells were co-treated with 100 nM PMA, and 25, 50, and 100 µg/mL AGE for 3 h. Cell viability was evaluated using the WST-1 assay, and NETosis was quantified using SYTOX Green. (**B**) MPO and NE production in the culture supernatants were measured using ELISA. (**C**) dHL-60 cells were co-treated with 100 nM PMA, and 25, 50, and 100 µg/mL AGE for 20 min, following which the cells were stained with DHR123 to assess ROS production. (**D**) dHL-60 cells were co-treated with 100 nM PMA, and 25, 50, and 100 µg/mL AGE for 3 h and (**E**) 20 min, then visualized using confocal and fluorescence microscopy. (**F**) The mRNA expression levels of NETs-related enzymes, (**G**) inflammatory cytokine and chemokines-related markers, and (**H**) neutrophils maturation and migration-related genes were measured using quantitative real-time polymerase chain reaction. Data were analyzed via one-way ANOVA by Dunnett’s test. All values have been reported as mean ± SD. * *p* < 0.05, ** *p* < 0.01, *** *p* < 0.001.

AGE suppressed the inflammatory response due to immune cell involvement, particularly the levels of NETosis markers and neutrophil recruitment in BALF (Figure 1). Based on these findings, we investigated the effects of AGE on neutrophil activation in PMA-stimulated dHL-60 cells. To investigate whether AGE affects the viability of dHL-60 cells, we treated the cells with AGE (25, 50, and 100 µg/mL) and subjected them to the WST-1 assay. No cytotoxicity was observed at any AGE concentration tested (Figure 4A). We evaluated the inhibitory effect of AGE on NETosis, a process by which neutrophils release NETs. NETosis was markedly increased in PMA-stimulated dHL-60 cells; however, these levels were inhibited in AGE-treated dHL-60 cells in a dose-dependent manner. Next, we measured the levels of NE and MPO, the two key markers of NETs. PMA-stimulated dHL-60 cells showed increased NE and MPO production; however, in AGE-treated dHL-60 cells, these levels decreased in a dose-dependent manner (Figure 4B). Furthermore, the release of DNA from cells and MPO levels in NETs structures were evaluated using confocal microscopy (Figure 4D). Consistent with the results shown in Figure 4B, MPO was released extracellularly from PMA-stimulated dHL-60 cells. However, this release was inhibited in AGE-treated cells at 100 g/mL, with the MPO levels similar to those in control cells. The ability of neutrophils to generate ROS is crucial for forming NETs. DHR123, a fluorescent indicator of cytosolic ROS, was used to evaluate cytosolic ROS production. As described in Figure 4C,E, ROS production was markedly increased in PMA-stimulated dHL-60 cells, whereas ROS production was decreased in AGE-treated dHL-60 cells. As shown in Figure 4F, the mRNA levels of MPO, ELANE, deoxyribonuclease 1, and peptidylarginine deiminase 4 were increased in PMA-stimulated dHL-60 cells; however, this increase was significantly suppressed in AGE-treated cells. Next, we showed the mRNA levels of C-X-C Motif chemokine receptor 1 (CXCR-1), CXCR-2, CXCR-4, and interleukin 1β, known to play a critical role in the regulation of ROS production and NETs formation in neutrophils, were increased in PMA-stimulated dHL-60; however, when co-treated with AGE, the mRNA levels of these significantly decreased (Figure 4G). HMGB, RUNX1, and KLF6 have been identified as transcription factors that regulate neutrophil maturation and migration to sites of inflammation and injury, including ITGAL and CX3CR-1 [35]. The mRNA levels of HMGB, RUNX1, and KLF6 increased in PMA-stimulated dHL60 cells and decreased in AGE-treated cells. In addition, the increase in mRNA levels of ITGAL and CX3CR-1 in PMA-stimulated cells was downregulated in AGE-treated cells. These results suggested that AGE inhibited both ROS production and NET formation in PMA-stimulated dHL-60 cells.

## 4. Discussion

Elevated PM levels are found in polluted atmospheres throughout the worldwide, and exposure of PM causes hazardous effects that result in premature death, including stroke, lung cancer, chronic obstructive pulmonary disease, and other pulmonary diseases. PM comprises SO_2_, NO_3_, NH_4_, organic carbon, and sodium ions as the main chemical components, and bezno[α]pyrene, NO_2_-, and nickel in PM are known to be harmful to human health. However, the most important factors to consider in the risk of PM are the origin of the substances constituting it. PM components are affected by various environmental factors, such as vehicles, construction sites, and the burning of fuels, such as wood, heating oil, and natural sources [36], and various PMs have different characteristic effects. For example, both urban- and desert-derived PM aggravate lung eosinophilia by enhancing the Th2-immune response along with increasing M2 macrophages. However, the effect was greater in response to microbial element-rich, desert-derived PM than in response to organic, chemically rich, urban-derived PM. These results indicated that desert-derived PM may cause greater effects upon human respiratory health than urban-derived PM [37]. Therefore, the most crucial factor in PM is the type of PM used. In the present study, to select the PM sample, we tested the effects of five different types of PM in vitro and in vivo. In an in vitro experiment using MH-S alveolar macrophages and H292 airway epithelial cells, 1648a treatment resulted in the highest production of inflammatory cytokines, such as MIP-2 and IL-8. In in vivo mouse model, the treatment of 1648a showed the highest inflammatory responses in BALF, such as increased total cell counts and inflammatory cytokine and chemokine levels. Based on these results, we used NIST reference sample SRM 1648a in this study. Another critical point in research related to PM is that PM is generally classified as PM10, PM2.5, and PM0.1 according to size. The range of PM that can penetrate the respiratory tract varies depending on the size, and the extent to which the PM penetrates influences the inflammatory risk of PM. PM10 is deposited in the airway and penetrates the periphery of the lungs, whereas PM2.5 can penetrate deep into the lungs and alveoli. PM0.1 partially penetrates the body through the alveolar–capillary membrane, which separates air from the blood stream. It is reported that the 1648a size distribution was more than 50% and less than 2.5 µm [38]. In this study, 1648a treatment in an in vivo model penetrated the mouse lung and alveoli, and 1648a deposited in the lung tissues caused chronic inflammation and respiratory diseases. One way to solve this problem is to discover materials that can suppress PM-induced inflammation. The AGE found in this study can effectively control PM-induced respiratory inflammation in a concentration-dependent manner.

Dexamethasone, an adrenal corticosteroid, possesses anti-inflammatory and immunosuppressive properties. In our research, we used dexamethasone as a positive control at a concentration of 3 mg/kg [39], which significantly reduced PM-induced pulmonary inflammation. However, the long-term administration of dexamethasone can lead to side effects such as muscle weakness, vomiting, diarrhea, and infection, making it a challenging treatment option [40]. Consequently, there is a need to explore anti-inflammatory compounds without such side effects. In our study, we administered AGE to mice at concentrations of 100 and 200 mg/kg, and observed no toxicity or adverse effects. Furthermore, AGE significantly mitigated PM-induced pulmonary inflammation compared to dexamethasone, and this effect was found to be concentration-dependent. When converted for a 60 kg adult, these doses equate to a daily intake of 500 mg^−1^ g. This is considered a dosage that could potentially be developed as an alternative medicine.

*Artemisia gmelinii* Weber ex Stechm (AG) contains various bioactive compounds such as flavonoids, terpenoids, coumarins, caffeoylquinic acids, sterols, and acetylenes. In an earlier study, we demonstrated the beneficial effects of AGE in a respiratory disease model for COPD and allergic airway inflammation [20,41]. Additionally, the present study suggests that AGE effectively inhibits PM-induced lung damage and pulmonary inflammatory responses by suppressing the NF-κB/MAPK pathway and neutrophil infiltration. Therefore, AGE has been proven to be a functional material that can effectively ameliorate various respiratory diseases. Currently, AGE is undergoing a process involved in developing functional foods, and safety and standardization studies are being conducted alongside efficacy studies. As a result of a toxicity test to evaluate the safety of AGE, we estimated that the Approximate Lethal Dose (ALD) of a single oral dose of AGE in Sprague-Dawley rats was greater than 5000 mg/kg for both males and females. In the 13-week repeated-dose toxicity test, AGE was administered at doses of 625, 1250, and 2500 mg/kg/day, and no abnormal symptoms related to AGE exposure were observed. Based on these results, the No Observed Adverse Effect Level (NOAEL) of 2500 mg/kg/day was determined for both male and female rats. The genotoxicity test included chromosome aberration, micronucleus, and reverse mutation tests, and the results indicated that no abnormalities were observed in AGE. Therefore, we confirmed that AGE did not exhibit genotoxic effects or induce any detectable abnormalities in the genotoxicity test. Furthermore, in a standardization study, AGE established scopolin as a quality control index component for standardization purposes. As a result, we showed that scopolin content was 6.3 mg/g ± 0.2%, and standardization was maintained. Meanwhile, besides scopolin, functional components of AGE include scopoletin, chlorogenic acid, hyperoside, 4,5-di-O-caffeoylquinic acid, 3,5-di-O-caffeoylquinic acid, and 3,4-di-O-caffeoylquinic acid. Scopoletin was reported to inhibit the production of inflammatory cytokines such as tumor necrosis factor α (TNF-α), interleukin 6 (IL-6), and IL-8 in PMA and A23187-induced HMC-1 (Human mast cell Line) cells through the IκB/NF-κB signaling pathways [42]. Chlorogenic acid attenuates the inflammatory response of LPS-induced BV2 cells [43]. Hyperoside, a flavonoid glycoside isolated from A. gmelinii, has been shown to exhibit protective effects against liver damage in mice [44], and 3,5-di-O-caffeoylquinic acid has been found to lower adipocyte differentiation by enhancing HO-1 in 3T3-L1 cells [45]. Because the AGE used in this study was in an extract state, the direct mechanism of a specific functional component cannot be explained. However, based on the results of the present study, each functional component may contribute to the improvement of respiratory diseases in a complex or synergistic manner. In future studies, we plan to investigate the functions and mechanisms of each component.

Oxidative stress has been proposed as a critical mechanism underlying PM-induced inflammation and cytotoxicity [46]. Oxidative stress generated in the lungs is a defense mechanism that protects against lung injury. However, when exposed to a substance that causes lung damage, airway epithelial cells and alveolar macrophages initially respond and generate oxidative products; immune cells, such as neutrophils and inflammatory cells, migrate and infiltrate via secreted chemokines, resulting in the generation of oxidative products and the exacerbation of inflammatory responses. Therefore, the inhibition of oxidative stress caused by PM is vital for alleviating pulmonary diseases. ROS can activate downstream signaling pathways directly or indirectly in response to cellular factors, including the redox-sensitive MAPK signaling pathway and NF-κB signaling pathway [47]. The activation of NF-κB is one of the crucial transcriptional factors that can regulate the expression of COX-2 and various inflammatory cytokines and chemokines such as IL-6, IL-8, and TNFα in stimulated cells [48]. Furthermore, MAPK signaling plays a key role in chronic inflammation and p38 MAPK is phosphorylated by PM-induced ROS. In this study, 1648a treatment significantly increased the phosphorylation of p38 MAPK and the phosphorylation and translocation of NF-κB p65, and treatment with AGE effectively inhibited the phosphorylation of p38 MAPK and the phosphorylation and translocation of NF-κB p65, resulting in the suppression of inflammatory cytokines, chemokines, and COX-2 expression (Figure 1 and Figure 2). NF-κB and MAPK, well-known as pro-inflammatory signals, maintain homeostasis in balance with the NRF2 antioxidant signal [49]. NRF2 is a transcription factor critical for the induction or constitutive expression of antioxidant enzymes [49]. It has been reported that NRF2 plays a key protective role against lung injury by modulating the airway’s innate immune response triggered by oxidative stress from PM [50]. NRF2 binds to the Antioxidant Response Element (ARE) to regulate the transcription of antioxidant genes such as HO-1 [49]. Therefore, the NFR2/HO-1 signaling pathway is essential for confirming the antioxidant effects of AGE. As shown in Figure 2, AGE induced NRF2 expression in a dose-dependent manner. Based on these results, we suggest that AGE treatment induced the activation of the NRF2/HO-1 pathway and alleviated PM-induced inflammation by suppressing the phosphorylation of p38 MAPK and the phosphorylation and translocation of NF-κB pathway.

Neutrophilic inflammation is crucial in many pulmonary diseases, such as COPD, asthma, and cystic fibrosis [51]. In general, neutrophils, dominant circulating leukocytes that constitute approximately 70% of white blood cells, represent a key component of the innate immune system and are the first immune cells recruited to the lungs following pathogens. Airway neutrophilia is a characteristic observed in various lung pathologies, and patients with airway diseases often exhibit enhanced and more detrimental neutrophilic inflammation [51]. For instance, the number of neutrophils has been associated with airway obstruction, a decline in forced expiratory volume in one second, a decrease in gas transfer, and the development of emphysema [52]. Neutrophils utilize several host defense mechanisms, including phagocytosis, degranulation, ROS, reactive nitrogen species production, and pro-inflammatory cytokine production. In case of overwhelming infection or inflammation, neutrophils have been described as releasing their decondensed DNA in web-like structures called ‘NET’ outside the cell [53]. NETs are composed of decondensed chromatin fibers coated with antimicrobial granular and cytoplasmic proteins, such as myeloperoxidase, neutrophil elastase (NE), and α-defensins [54]. In particular, NE causes mucosal obstruction of the airways, a decrease in ciliary motility, and failure of capillary motility by upregulating the gene expression of secreted mucins in the airways, MUC5AC, and, finally, destroying lung tissues [55]. Taken together, controlling the neutrophil response is important for the prevention or treatment of pulmonary disease. In this study, we constructed an animal model of lung injury using 1648a and confirmed that mainly neutrophilic inflammatory responses occur. Based on this, we focused on neutrophils, and as a result, it was confirmed that AGE inhibited neutrophil infiltration in both BALF and lung tissues in the PM-induced lung injury model and suppressed NETosis, known as an exacerbating factor in pulmonary diseases (Figure 1 and Figure 3). In addition, it was confirmed that NE, MPO, and ROS were suppressed by AGE treatment through the inhibition of NETosis-related factors such as transcription factors and chemokine receptors (Figure 4). Therefore, we suggest that PM exposure can cause lung injury accompanied by neutrophilic inflammation, and the AGE presented in this study may help prevent inflammatory responses and lung damage by regulating neutrophils.

Our results suggest that although AGE suppresses NF-κB signaling pathways (Figure 2 and Figure 3) and thus suppresses overall chemokine production, more detailed further studies including transcriptional regulation are needed on the mechanism of AGE-mediated suppression of CXCL-1 or RANTES. For example, the expression of CXCL-1 is regulated by the activation of NF-κB, mediated by IL-1β, IL17R, TRAF6, and TAK1, and is mainly produced in lung fibroblasts, bronchial epithelial cells, and resident macrophages in lung inflammation models [56]. Additionally, RANTES is mainly produced by either T lymphocytes or epithelial cells and promotes the recruitment of monocytes, macrophages, and lymphocytes [57]. Similar to recent transcriptomic analyses about neutrophils [35], our current observations also revealed that AGE might directly modulate various inflammatory and immune programs of neutrophils mediated by transcriptional regulation of HMGB, RUNX1, and KLF6. Therefore, we planned further studies for identifying transcriptional regulation of inducible chemokine expressions modulated by AGE requires the coordinated regulation of multiple transcription factors in diverse immune cells such as epithelial cells, macrophages, and lymphocytes in the lung inflammation models.

## 5. Conclusions

In conclusion, oral administration of AGE attenuated PM-induced lung injury symptoms, including the infiltration of inflammatory cells, the increase in chemokines, mucus hypersecretion, and damage to lung parenchyma. Furthermore, AGE suppressed the production of inflammatory mediators by inhibiting the phosphorylation of p38 MAPK and the phosphorylation and translocation of NF-κB p65 via the activation of the NRF2/HO-1 pathway in PM-stimulated lung tissues. In particular, concordant with in vivo results, the attenuating effect of AGE on pulmonary inflammation was also confirmed in neutrophils. AGE suppressed the NETs formation by inhibiting ROS production in HL-60 cells. Therefore, AGE administration may be an effective approach for treating or preventing PM-induced lung injury.

## Data Availability

The data presented in this study are available in the article and Supplementary Material.

## Supplementary Materials

The following supporting information can be downloaded at: https://www.mdpi.com/article/10.3390/antiox12081591/s1, Table S1: Primers used for real-time quantitative PCR. Figure S1: Effect of AGE on phosphorylation of p38 in the lung tissues.

## Abbreviations

The following abbreviations are used in this manuscript.

AG	Artemisia gmelinii Weber ex Stechm
AGE	Artemisia gmelinii Weber ex Stechm ethanol extract
ALI	Acute lung injury
BALF	Bronchoalveolar lavage fluid
COPD	Chronic Obstructive Pulmonary Disease
COX-2	Cyclooxygenase-2
CS	Cigarette smoke
CSE	Cigarette smoke extract
CXCL-1	C-X- motif chemokine ligand 1
CXCR-1	C-X-C Motif chemokine receptor 1
CXCR-2	C-X-C Motif chemokine receptor 2
CXCR-4	C-X-C Motif chemokine receptor 4
CX3CR-1	C-X3-C Motif chemokine receptor 1
DAB	3,3′-diaminobenzidine
DAPI	4′,6-diamidine-2′-phenylindole dihydrochloride
dHL-60	Differentiated HL-60
DHR123	Dihydrorhodamine 123
HBSS	Hank’s balanced salt solution
HMGB1	High mobility group box 1
HO-1	Heme Oxygenase-1
H&E	Hematoxylin and eosin
IHC	Immunohistochemistry
IL-1β	Interleukin-1β
ITGAL	Integrin Alpha L
KLF6	Kruppel-like factor 6
LPS	Lipopolysaccharide
MAPK	Mitogen-activated protein kinase
MCP-1	Monocyte chemoattractant protein-1
MIP-1α	Macrophage Inflammatory Protein-1α
MPO	Myeloperoxidase
NE	Neutrophil elastase
NETs	Neutrophil extracellular traps
NF-κB	Nuclear factor kappa-light-chain-enhancer of activated B cells
NIST	National Institute of Standard and Technology
NRF2	Nuclear factor erythroid-2-related factor 2
PADI4	Peptidylarginine Deiminase 4
PAHs	Polycyclic aromatic hydrocarbons
PAS	Periodic acid–Schiff
PBS	Phosphate-buffered saline
PM	Particulate matter
PMA	Phorbol 12-myristate 13-acetate
RANTES	Regulated on activation, Normal T cell Expressed and Secreted
ROS	Reactive oxygen species
RUNX1	Run-related transcription factor 1
TARC	Thymus and Activation-Regulated Chemokine
WHO	World Health Organization
WST-1	Water-Soluble Tetrazolium 1
