# Supplementary material for: Artemisia gmelinii Extract Attenuates Particulate Matter-Induced Neutrophilic Inflammation in a Mouse Model of Lung Injury
Source: Antioxidants (Basel). 2023 Aug 9;12(8):1591. doi: 10.3390/antiox12081591 (PMC10451698; doi:10.3390/antiox12081591)
Supplement: Supplementary file 1 [file antioxidants-12-01591-s001.zip › antioxidants-2440525-for publish-supplementary.pdf]

Supplementary Table S1. Primers used for real-time quantitative PCR.

Genes		Sequences (5'-3')
Hu_MPO	F:	AGCAGGACAAATACCGCACCA
	R:	AGAGAAGCCGTCCTCATACTCC
Hu_ELANE	F:	TGCGCCCAACTTCGTCATGTCG
	R:	CGTAGCCGTTTTCGAAGATGCG
Hu_DANSE1	F:	CCAGACACCTATCACTACGTGG
	R:	CTCTCGGTTGAAGGTGTCGTTC
Hu_PADI4	F:	GCACAACATGGACTTCTACGTGG
	R:	CACGCTGTCTTGGAACACCACA
Hu_IL1β	F:	CCACAGACCTTCCAGGAGAATG
	R:	GTGCAGTTCAGTGATCGTACAGG
Hu_CXCR1	F:	TCCTTTTCCGCCAGGCTTACCA
	R:	GGCACGATGAAGCCAAAGGTGT
Hu_CXCR2	F:	TCCGTCACTGATGTCTACCTGC
	R:	TCCTTCAGGAGTGAGACCACCT
Hu_CXCR4	F:	CTCCTCTTTGTCATCACGCTTCC
	R:	TCCTTCAGGAGTGAGACCACCT
Hu_HMGB1	F:	GGATGAGGACACTGCTGTAGAG
	R:	GTCCTTGAACTTCTTTTTGGTCTC
Hu_RUNX1	F:	CCACCTACCACAGAGCCATCAA
	R:	TTCACTGAGCCGCTCGGAAAAG
Hu_KLF6	F:	AACCAGGCACTTCCGAAAGCAC
	R:	CTCAGAGGTGCCTCTTCATGTG
Hu_ITGAL	F:	CTGCTTTTGCCAGCCTCTCTGT
	R:	GCTCACAGGTATCTGGCTATGG
Hu_CX3CR1	F:	CACAAAGGAGCAGGCATGGAAG
	R:	CAGGTTCTCTGTAGACACAAGGC

Supplementary Figure S1

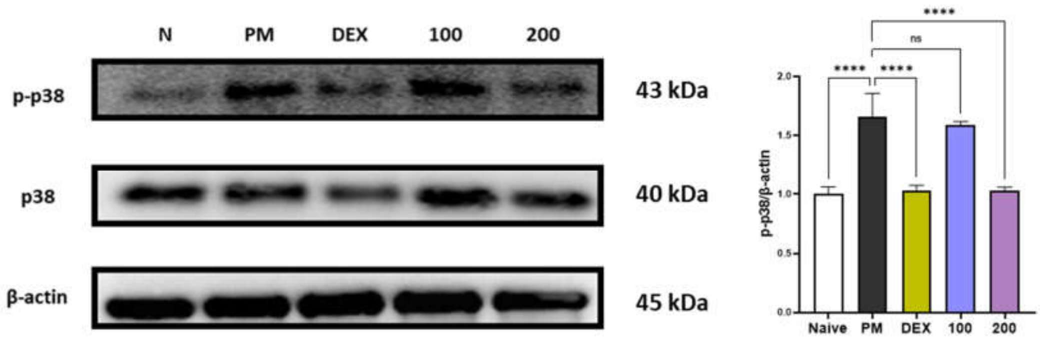

Figure S1. Effect of AGE on phosphorylation of p38 in the lung tissues. Protein expression levels of p-p38, p38, and β-actin. Data were analyzed using one-way ANOVA, followed by Dunnett's test. All values have been reported as mean ± SD. * $p < 0.05$, ** $p < 0.01$, *** $p < 0.001$, **** $p < 0.0001$.
